# Effect of Nurture on Nature Through Platelet Serotonin and Dopamine

**DOI:** 10.1192/bjo.2025.10728

**Published:** 2025-06-20

**Authors:** Devasis Ghosh

**Affiliations:** Health Point Hospital, Kolkata, India

## Abstract

**Aims:** Each person is born with an unique personality/mental nature, determined by the genetic predisposition from biological parents. Starting from intrauterine period till the last date of life human beings are subjected to innumerable stressful factors, for which we are neither prepared or trained to face. Some of these stressors may have detrimental effects on our behavioural patterns by influencing the levels of neurotransmitters mainly platelet serotonin and dopamine (which has an inverse relationship with serotonin).

**Methods:** Here we present a case of ‘SS’, a Muslim lady 33 years married to a staunch Hindu male after having an affair for 6 years presenting in the OPD with recurrent suicidal thoughts for last 4 months with one failed attempt. She comes from a broken family. From her childhood she had seen her father regularly abusing her mother verbally and physically. Her mother separated when she was just 15 years and remarried. After one year of separation SS lost her biological father in a train accident which had affected her greatly.

After that loss, she ran away from her mom and stepfather and was staying alone when she met her present husband. After marriage her husband was also found to be very abusive verbally and physically and did not allow her to eat non-veg, neither was she allowed to do her regular namaz prayers. Recently she found her husband having an extra-marital affair that triggered the suicidal attempt. Her platelet serotonin was found to be very low and she was prescribed SSRI and antipsychotics.

**Results:** Leaving aside the natural calamities, aberrant/unsocial behaviours in the society mostly go unnoticed or not given the due importance until a grave crime is committed or the victims who are subjected to these sort of behaviours, by some reason or rather, themselves develop mental health issues.

Thus the preventable cause and effect factors are usually overlooked and treatment is targeted only to the affected patients. This causes a huge gap in the management of mental health issues in the society at large which seems to be increasing day by day.

**Conclusion:** Routine platelet serotonin test may help to unearth the hidden players with no insight causing unsocial and maleficent behaviours and thus affecting unwary family members or anyone outside coming in contact with them. Until and unless we cater to these predisposing and/or precipitating factors leading to mental stress, good mental health of the global society remains a myth.

*No financial sponsorship was taken in this case study.

